# VirPipe: an easy-to-use and customizable pipeline for detecting viral genomes from Nanopore sequencing

**DOI:** 10.1093/bioinformatics/btad293

**Published:** 2023-05-02

**Authors:** Kijin Kim, Kyungmin Park, Seonghyeon Lee, Seung-Hwan Baek, Tae-Hun Lim, Jongwoo Kim, Balachandran Manavalan, Jin-Won Song, Won-Keun Kim

**Affiliations:** Chair for Clinical Bioinformatics, Saarland University, Saarbrücken 66123, Germany; Department of Biomedical Sciences, BK21 Graduate Program, Korea University College of Medicine, Seoul 02841, Republic of Korea; Department of Microbiology, Korea University College of Medicine, Seoul 02841, Republic of Korea; Department of Microbiology, College of Medicine, Hallym University, Chuncheon 24252, Republic of Korea; Institute of Medical Research, College of Medicine, Hallym University, Chuncheon 24252, Republic of Korea; Department of Microbiology, College of Medicine, Hallym University, Chuncheon 24252, Republic of Korea; Department of Biomedical Sciences, BK21 Graduate Program, Korea University College of Medicine, Seoul 02841, Republic of Korea; Department of Microbiology, Korea University College of Medicine, Seoul 02841, Republic of Korea; Department of Integrative Biotechnology, College of Biotechnology and Bioengineering, Sungkyunkwan University, Suwon 16419, Republic of Korea; Department of Biomedical Sciences, BK21 Graduate Program, Korea University College of Medicine, Seoul 02841, Republic of Korea; Department of Microbiology, Korea University College of Medicine, Seoul 02841, Republic of Korea; Department of Microbiology, College of Medicine, Hallym University, Chuncheon 24252, Republic of Korea; Institute of Medical Research, College of Medicine, Hallym University, Chuncheon 24252, Republic of Korea

## Abstract

Detection and analysis of viral genomes with Nanopore sequencing has shown great promise in the surveillance of pathogen outbreaks. However, the number of virus detection pipelines supporting Nanopore sequencing is very limited. Here, we present VirPipe, a new pipeline for the detection of viral genomes from Nanopore or Illumina sequencing input featuring streamlined installation and customization.

**Availability and implementation:**

VirPipe source code and documentation are freely available for download at https://github.com/KijinKims/VirPipe, implemented in Python and Nextflow.

## 1 Introduction

Nanopore sequencing, one of the third-generation high-throughput sequencing (HTS) technologies, has been widely applied in the identification and discovery of pathogens. Featured with real-time and on-site sequencing, it has been applied in metagenomic approaches, whole-genome sequencing for epidemiological surveillance, and genomic characterization and identification of putative pathogens.

Although many virus detection pipelines have been developed to automate the detection of viral reads and the reconstruction of viral genomes from HTS input thus far, only a few support Nanopore sequencing because of its relatively short history. As shown in Supplementary Table S1 of Supplementary File S1, three virus detection pipelines support Nanopore input. However, these have weaknesses that hamper their active use in research. GenomeDetective (Vilsker et al. 2019) limits the number of analyses at a time and cannot be utilized offline in a free version. NanoSPC (Xu et al. 2020) is not in service as of February 2023. Vir-MinION (Mastriani et al. 2022) requires users to install all of the component programs manually, which is demanding for users unskilled at handling Unix-like OS.

One can consider using general metagenome binning pipelines listed in Supplementary Table S2 of Supplementary File S1. However, they also require formidable installation steps and downloads of large database files because they typically address all microbiomes not limited to viruses.

In this regard, an easy-to-use pipeline is urgently needed to fulfil the rising demand for analysis with Nanopore sequencing input in relevant fields.

Here, we present VirPipe, a bioinformatics pipeline for virus identification and discovery with Nanopore or Illumina sequencing input. We have focused on developing a user-friendly and customizable pipeline so that it can be accessible by a wide range of users from novices to experts. Furthermore, it is equipped with three distinct analysis methods: reference mapping, taxonomic classification, and contig analysis. These methods complement each other and result in a comprehensive analysis.

## 2 Materials and methods

### 2.1 Workflow summary

**Figure 1.**
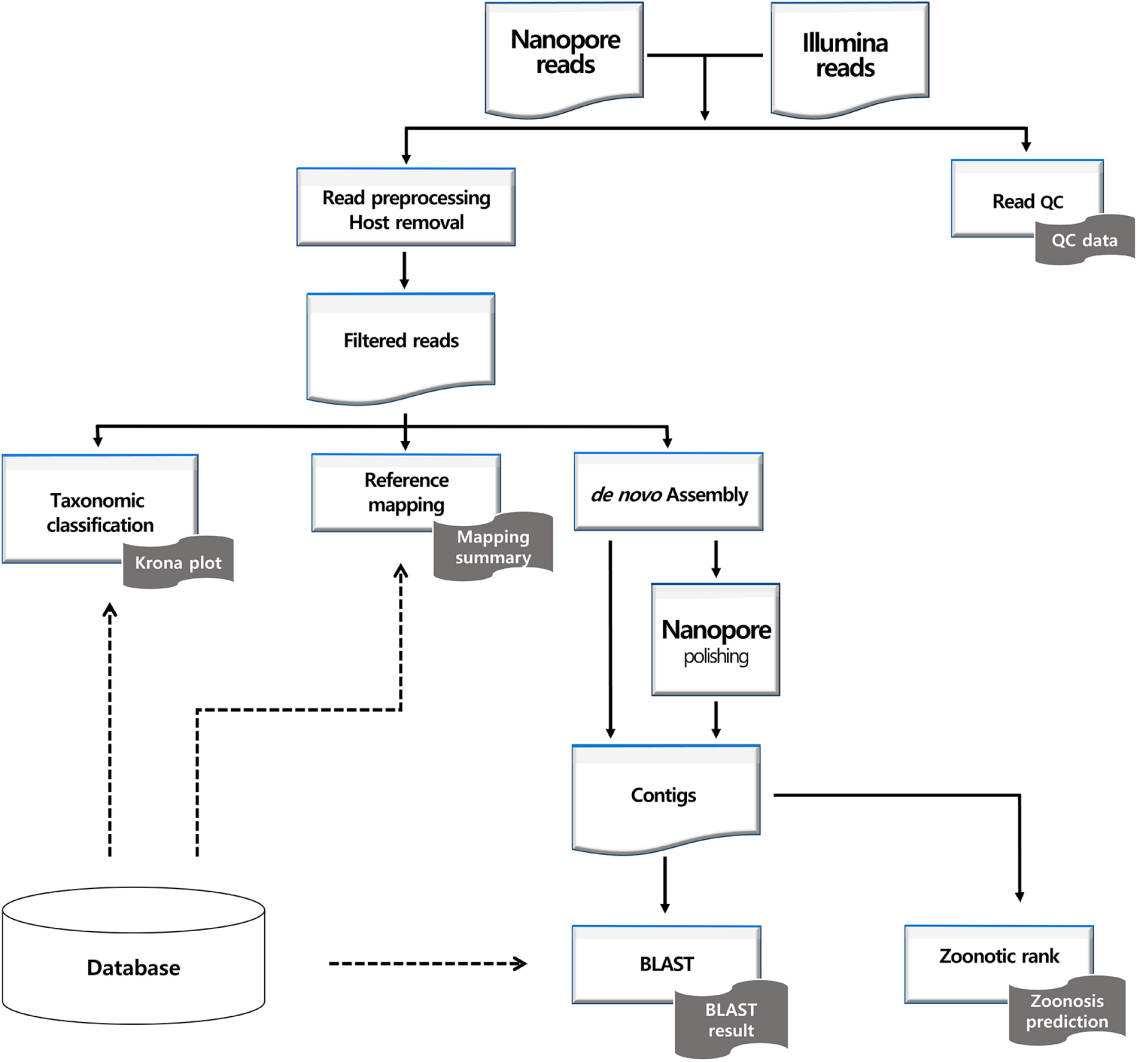
VirPipe workflow.

Figure 1 shows the VirPipe workflow. First, sequencing reads are filtered by the average base quality and read length. Additionally, host-derived reads can be removed by mapping the reads to the host genome. Then, the remaining reads are given as an input to the main analysis modules.

The reference mapping module maps the reads onto each given viral genome with Minimap2 (Li 2018), and the mapping results are organized into a more comprehensible report by Qualimap (García-Alcalde et al. 2012).

In the taxonomic classification module, the reads are classified into taxonomies by Centrifuge (Kim et al. 2016) or Kraken2 (Wood et al. 2019) for Nanopore or Illumina reads, respectively. Finally, contigs are *de novo* assembled by Flye (Kolmogorov et al. 2019) or SPAdes (Bankevich et al. 2012) with Nanopore or Illumina reads, respectively. The additional polishing step is performed only for contigs made from Nanopore reads in order to correct errors derived from its low sequencing accuracy. The assembled contigs’ closest references are found using BLAST+ (Camacho et al. 2009). Optionally, the potential zoonosis of the contigs can be estimated by the Zoonotic rank (Mollentze et al. 2021).

### 2.2 Software implementation

To make the pipeline easier to use, we hid the programmatic details from the viewpoint of the user and set plausible defaults to most parameters. But users can customize the pipeline by changing the parameters and skipping some steps. Also, each step can be run independently with initial input or intermediate files. Each pipeline step is run by a Nextflow code that is wrapped by a Python script, providing a more user-friendly interface. Using the Docker containers technology integrated with Nextflow, the pipeline can be easily installed in an internet-connected environment. The output directory includes raw output files from every analysis step.

## 3 Use case

To demonstrate its utility, we ran VirPipe with published sequencing datasets. The list of sample datasets can be found in Supplementary File S2.

The raw output files can be compiled into a well-organized analysis report. For example, we generated a sample analysis report of SRR22029862 from Park et al. (2021) attached in Supplementary File S3. This dataset includes Nanopore reads sequenced from the lung tissue of a rodent whose library was amplified via multiplex polymerase chain reaction targeting *Hantaan orthohantavirus* (HTNV). Experiments have confirmed that the tissue was HTNV positive.

As seen in the report, the results of all three analysis modules point out that there exist HTNV-related reads in the input reads. In the reference mapping, all three segments of HTNV were almost entirely covered by the input reads. Also, in the taxonomic classification, a majority of the reads were classified into HTNV. Finally, a lot of assembled contigs showed high similarity with HTNV reference sequences in blast results generated from the contig analysis.

The raw output files from sample runs for other viruses can be found in Supplementary Data S4.

## Supplementary Material

btad293_Supplementary_Data

## Data Availability

The data underlying this article are available in the article and in its online supplementary material.
