# Supplementary material for: VirPipe: an easy-to-use and customizable pipeline for detecting viral genomes from Nanopore sequencing
Source: Bioinformatics. 2023 May 2;39(5):btad293. doi: 10.1093/bioinformatics/btad293 (PMC10191607; doi:10.1093/bioinformatics/btad293)
Supplement: btad293_Supplementary_Data [file btad293_supplementary_data.zip › btad293_Supplementary_Data/Supplementary file 1. Tables of published pipelines.docx]

**Table 1. List of published virus detection pipelines**

| **Tool** | **VIP** | **VirusDetect** | **Genome**  **Detective** | **NanoSPC** | **ViroMatch** | **Vir-MinION** |
| --- | --- | --- | --- | --- | --- | --- |
| **Published year** | 2016 | 2017 | 2019 | 2020 | 2021 | 2022 |
| **Application type** | stand-alone | stand-alone online | online | stand-alone | stand-alone | stand-alone |
| **Long read-only support** | explicitly no | explicitly no | explicitly yes | explicitly yes | explicitly no | explicitly yes |
| **Freeware** | yes | yes | partially yes* | yes | yes | yes |
| **In service** | yes | yes | yes | No | yes | yes |

* The free service allows only one analysis at a time

**Table 2. List of published metagenome binning pipelines**

| **Tool** | **EDGE** | **SqueezeMeta** | **ATLAS** | **Muffin** | **nf-core/mag** |
| --- | --- | --- | --- | --- | --- |
| **Published year** | 2017 | 2019 | 2020 | 2021 | 2022 |
| **Application type** | stand-alone online | stand-alone | stand-alone | stand-alone | stand-alone |
| **Long read-only support** | explicitly yes | explicitly yes | explicitly no | explicitly no | explicitly no |
| **Freeware** | yes | yes | yes | yes | yes |
| **In service** | yes | yes | yes | yes | yes |
