# Supplementary material for: VirPipe: an easy-to-use and customizable pipeline for detecting viral genomes from Nanopore sequencing
Source: Bioinformatics. 2023 May 2;39(5):btad293. doi: 10.1093/bioinformatics/btad293 (PMC10191607; doi:10.1093/bioinformatics/btad293)
Supplement: btad293_Supplementary_Data [file btad293_supplementary_data.zip › btad293_Supplementary_Data/Supplementary file 3. Sample analysis report.pdf]

Supplementary file 3. Sample analysis report

This sample report shows how raw output files generated by VirPipe can be compiled into a well-organized report. The report contains the output of VirPipe run with input Nanopore reads deposited in NCBI SRA with accession id SAMN31432881. Sample runs can be reproduced by following the instructions at https://github.com/KijinKims/VirPipe/wiki/Reproducing-sample-results.

General summary	
Mean read length	493.7
Mean read quality	10.4
Median read length	418.0
Median read quality	10.4
Number of reads	253,256.0
Read length N50	584.0
STDEV read length	325.2
Total bases	125,035,665.0
Number, percentage and megabases of reads above quality cutoffs	
>Q5	253252 (100.0%) 125.0Mb
>Q7	252802 (99.8%) 124.9Mb
>Q10	144217 (56.9%) 64.7Mb
>Q12	46914 (18.5%) 19.3Mb
>Q15	958 (0.4%) 0.2Mb
Top 5 highest mean basecall quality scores and their read lengths	
1	35.3 (5)
2	34.0 (8)
3	33.9 (3)
4	31.4 (16)
5	29.4 (3)
Top 5 longest reads and their mean basecall quality score	
1	4929 (8.0)
2	4528 (8.4)
3	4411 (7.8)
4	4388 (8.7)
5	4383 (8.2)

Table 1. descriptive statistics of initial input reads from QC plot generated from qc step.

Minimum read quality = 8, Minimum read length = 200	
Before preprocessing	After preprocessing
253256	209027

Table 2. The number of reads before/after preprocessing step.

Minimum read quality = 8, Minimum read length = 200	
Before preprocessing	After preprocessing
209027	208917

Table 3. The number of reads before/after remove-host step.

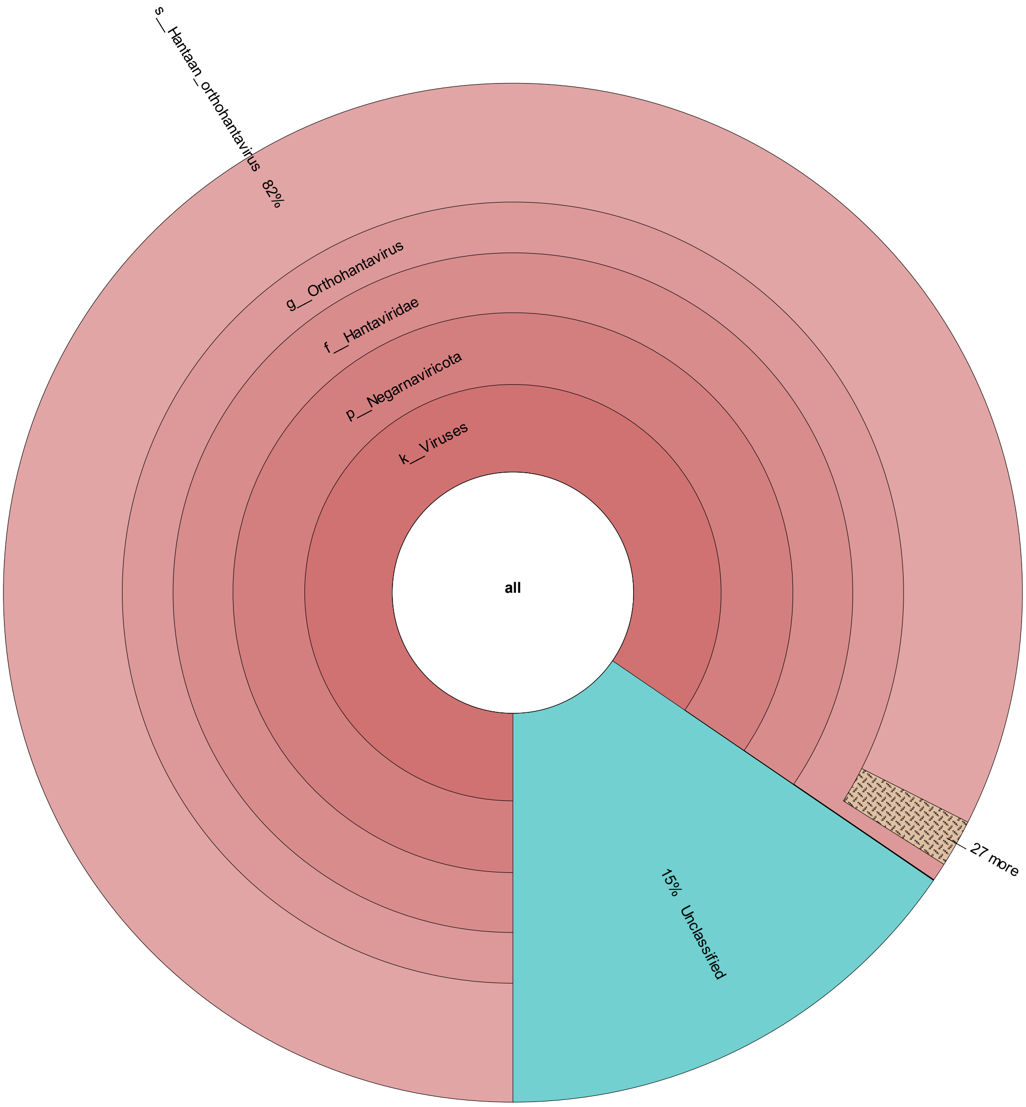

Figure 1. Krona chart generated in classify-taxonomy step.

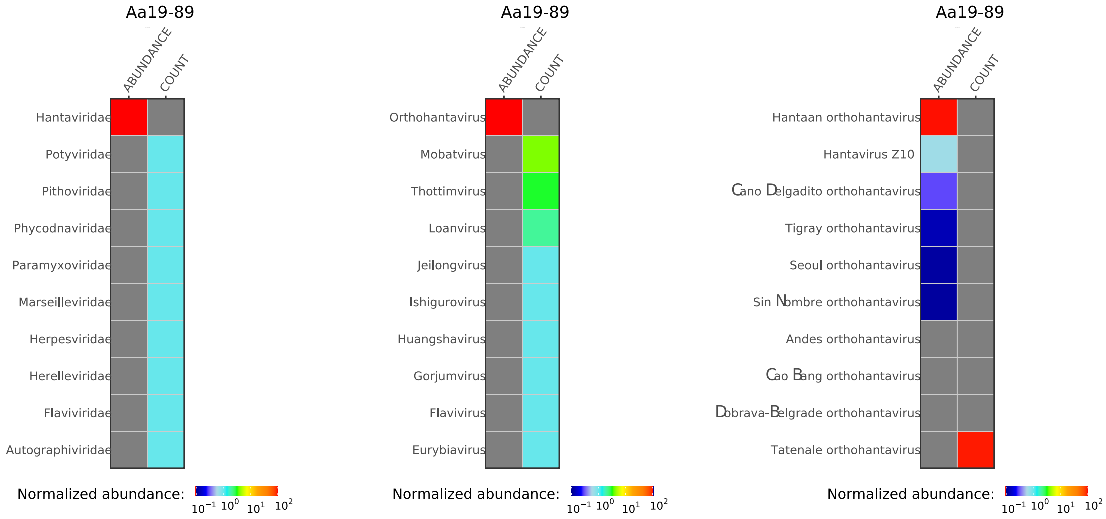

Figure 2. Heatmaps generated from the classify-taxonomy step.

From left to right, each indicates the classification result on the level of family, genus, and species.

SEQ_NAME	LEN	N_READS	N_COVERED_BASES	PERCENT_COVERED	AVG_COV	AVG_BASEQ	AVG_MAPQ
M14626.1	1696	54003	1696	100	14806.9	19	54.8
NC_005219.1	3616	76445	3615	99.9723	8676.46	19.3	50.9
NC_005222.1	6533	89263	6346	97.1376	4656.72	19.3	48.7
NC_038939.1	6559	306	1082	16.4964	17.3414	21.1	6.39

Table 4. summary of mapping results from map step. For the top three entries, from top to bottom, each corresponds to the reference sequence of the L, M, and S segment of *Hantaan Orthohantavirus* (HTNV). The last entry corresponds to the reference sequence of the L segment of *Prospect Hill virus,* which belongs to the Family *Hantaviridae* along with HTNV.

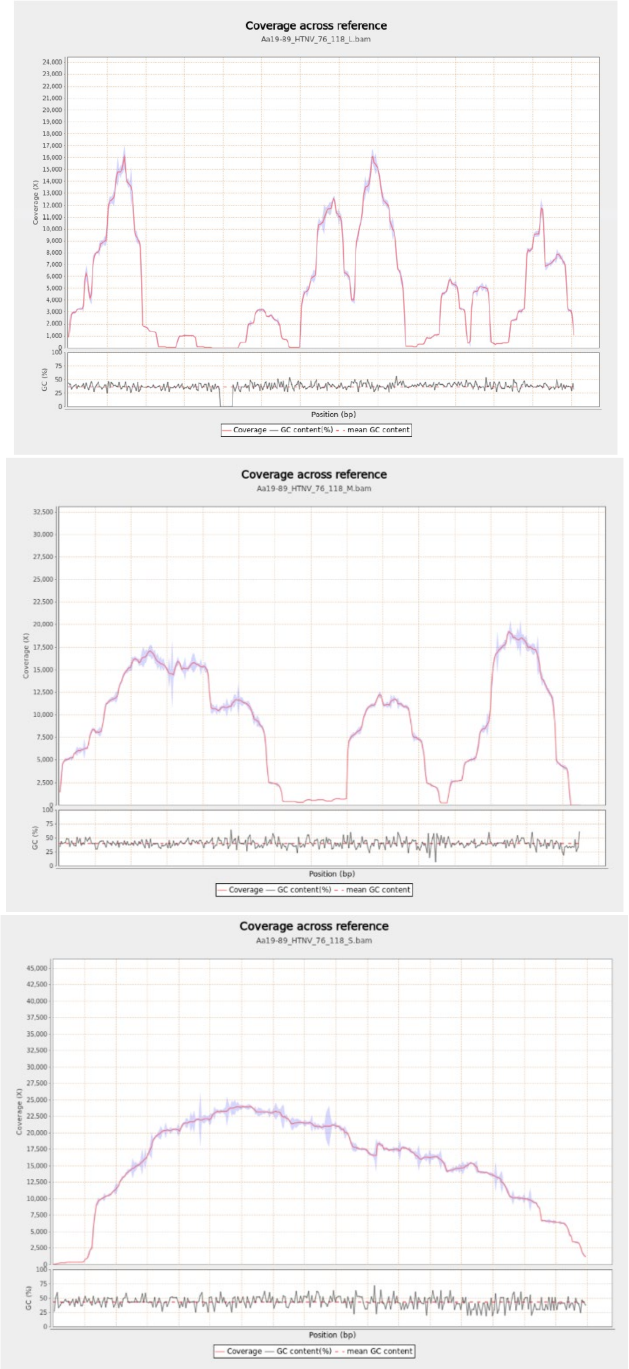

Figure 3. coverage across reference plots from Qualimap reports generated from map step. From top to bottom, each corresponds to the mapping result of input reads onto the reference sequence of the L, M, and S segment of *Hantaan Orthohantavirus* (HTNV).

<table>
<thead>
<tr><th>superkingdom</th><th>phylum</th><th>class</th><th>order</th><th>family</th><th>genus</th><th>species</th><th>TAX_ID</th><th>REF_ID</th><th>REF_TITLE</th><th>QUERY_ID</th><th>REF_ID.1</th><th>EVALUE</th><th>BITSCORE</th></tr>
</thead>
<tbody>
<tr><td>All</td><td>All</td><td>All</td><td>All</td><td>All</td><td>All</td><td>All</td><td>All</td><td>All</td><td>All</td><td>All</td><td>All</td><td>All</td><td>All</td></tr>
<tr><td>Viruses</td><td>Negarnaviricota</td><td>Ellioviricetes</td><td>Bunyavirales</td><td>Hantaviridae</td><td>Orthohantavirus</td><td>Hantaan orthohantavirus</td><td>1980471</td><td>gi|38371710|ref|NC_005218.1|</td><td>Hantaan virus, complete genome</td><td>tig13</td><td>gi|38371710|ref|NC_005218.1|</td><td>0</td><td>2316</td></tr>
<tr><td>Viruses</td><td>Negarnaviricota</td><td>Ellioviricetes</td><td>Bunyavirales</td><td>Hantaviridae</td><td>Orthohantavirus</td><td>Hantaan orthohantavirus</td><td>1980471</td><td>gi|38371712|ref|NC_005219.1|</td><td>Hantaan virus, complete genome</td><td>tig17</td><td>gi|38371712|ref|NC_005219.1|</td><td>0</td><td>2124</td></tr>
<tr><td>Viruses</td><td>Negarnaviricota</td><td>Ellioviricetes</td><td>Bunyavirales</td><td>Hantaviridae</td><td>Orthohantavirus</td><td>Hantaan orthohantavirus</td><td>1980471</td><td>gi|38371710|ref|NC_005218.1|</td><td>Hantaan virus, complete genome</td><td>tig60</td><td>gi|38371710|ref|NC_005218.1|</td><td>0</td><td>1914</td></tr>
<tr><td>Viruses</td><td>Negarnaviricota</td><td>Ellioviricetes</td><td>Bunyavirales</td><td>Hantaviridae</td><td>Orthohantavirus</td><td>Hantaan orthohantavirus</td><td>1980471</td><td>gi|38371710|ref|NC_005218.1|</td><td>Hantaan virus, complete genome</td><td>tig40</td><td>gi|38371710|ref|NC_005218.1|</td><td>0</td><td>1801</td></tr>
<tr><td>Viruses</td><td>Negarnaviricota</td><td>Ellioviricetes</td><td>Bunyavirales</td><td>Hantaviridae</td><td>Orthohantavirus</td><td>Hantaan orthohantavirus</td><td>1980471</td><td>gi|38371716|ref|NC_005222.1|</td><td>Hantaan virus segment L, complete genome</td><td>tig46</td><td>gi|38371716|ref|NC_005222.1|</td><td>0</td><td>1751</td></tr>
<tr><td>Viruses</td><td>Negarnaviricota</td><td>Ellioviricetes</td><td>Bunyavirales</td><td>Hantaviridae</td><td>Orthohantavirus</td><td>Hantaan orthohantavirus</td><td>1980471</td><td>gi|38371710|ref|NC_005218.1|</td><td>Hantaan virus, complete genome</td><td>tig1</td><td>gi|38371710|ref|NC_005218.1|</td><td>0</td><td>1681</td></tr>
<tr><td>Viruses</td><td>Negarnaviricota</td><td>Ellioviricetes</td><td>Bunyavirales</td><td>Hantaviridae</td><td>Orthohantavirus</td><td>Hantaan orthohantavirus</td><td>1980471</td><td>gi|38371710|ref|NC_005218.1|</td><td>Hantaan virus, complete genome</td><td>tig5</td><td>gi|38371710|ref|NC_005218.1|</td><td>0</td><td>1657</td></tr>
<tr><td>Viruses</td><td>Negarnaviricota</td><td>Ellioviricetes</td><td>Bunyavirales</td><td>Hantaviridae</td><td>Orthohantavirus</td><td>Hantaan orthohantavirus</td><td>1980471</td><td>gi|38371712|ref|NC_005219.1|</td><td>Hantaan virus, complete genome</td><td>tig34</td><td>gi|38371712|ref|NC_005219.1|</td><td>0</td><td>1648</td></tr>
<tr><td>Viruses</td><td>Negarnaviricota</td><td>Ellioviricetes</td><td>Bunyavirales</td><td>Hantaviridae</td><td>Orthohantavirus</td><td>Hantaan orthohantavirus</td><td>1980471</td><td>gi|38371710|ref|NC_005218.1|</td><td>Hantaan virus, complete genome</td><td>tig64</td><td>gi|38371710|ref|NC_005218.1|</td><td>0</td><td>1646</td></tr>
<tr><td>Viruses</td><td>Negarnaviricota</td><td>Ellioviricetes</td><td>Bunyavirales</td><td>Hantaviridae</td><td>Orthohantavirus</td><td>Hantaan orthohantavirus</td><td>1980471</td><td>gi|38371712|ref|NC_005219.1|</td><td>Hantaan virus, complete genome</td><td>tig17</td><td>gi|38371712|ref|NC_005219.1|</td><td>0</td><td>1607</td></tr>
</tbody>
</table>

Figure 4. partial result of megablast from blast step.
